# Epigallocatechin-3-gallate suppresses the growth of human osteosarcoma by inhibiting the Wnt/β-catenin signalling pathway

**DOI:** 10.1080/21655979.2022.2051805

**Published:** 2022-03-29

**Authors:** Chaoqun Dong, Zhigang Wang, Peng Shen, Yingguo Chen, Jinshu Wang, Hongbo Wang

**Affiliations:** a Department of General Surgery, Chongqing Traditional Chinese Medicine Hospital, Chongqing, P.R. China; b Department of Dermatology and Cosmetology, Chongqing Hospital of Traditional Chinese Medicine, Chongqing, P.R. China

**Keywords:** Osteosarcoma, epigallocatechin-3-gallate, anticancer effect, Wnt/β-catenin pathways

## Abstract

Osteosarcoma (OS) is one of the most common malignant tumors in adolescents. Due to local invasion, distant metastasis and drug resistance, the clinical treatment efficacy and prognosis of OS have remained almost unchanged for decades. Epigallocatechin-3-gallate (EGCG) is a unique catechin from tea leaves, and some studies have confirmed its antitumour effects on various tumors. Here, cellular experiments showed that EGCG significantly promoted OS cell apoptosis and inhibited proliferation, migration and invasion, and cell and animal experiments demonstrated that the Wnt/β-catenin pathway played an indispensable role in the antitumour effects of EGCG. Moreover, EGCG inhibited the growth of OS cells in vitro while suppressing tumor cell damage to the bone in situ and distant lung metastasis. The results indicate that the antitumour effect of EGCG on human OS may be mediated by regulating the Wnt/β-catenin pathway and that EGCG can be used alone or in combination with other regimens as a potentially effective anticancer treatment.

## Graphical Abstract

**Figure:**
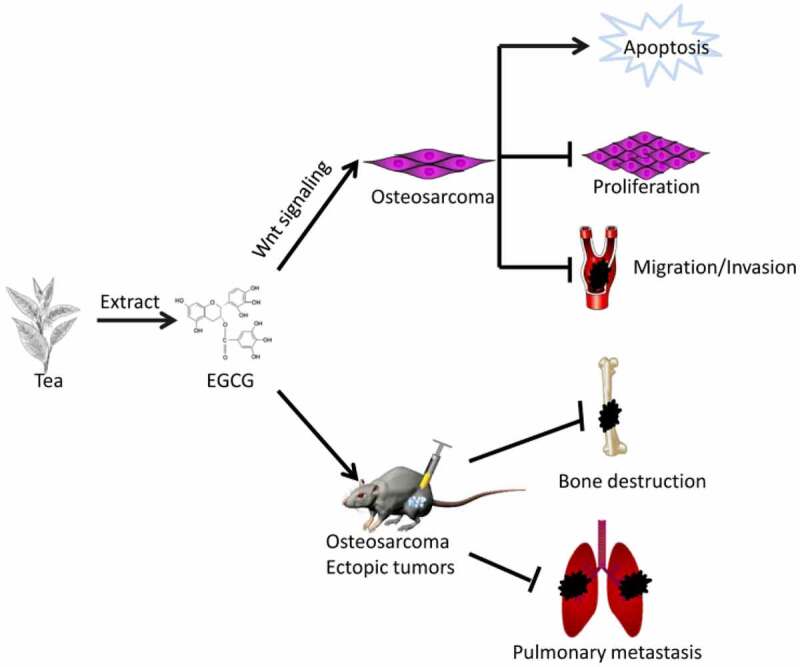

## Introduction

The most common malignant bone tumor in the skeletal system of children and adolescents is OS, which usually occurs in the metaphysis of the limbs [1]. Early detection and timely surgery and chemotherapy can effectively improve the prognosis of patients with OS, but most patients have difficulty tolerating the adverse effects of available chemotherapeutic drugs, and these severe adverse reactions often lead to a poor prognosis [1–3]. Surgical intervention combined with postoperative radiotherapy and chemotherapy remain the preferred treatments for OS, but due to issues such as tumor resistance, the severe side effects of chemotherapy, and early tumor metastasis, the therapeutic efficacy has stagnated for decades without significant improvements [4]. To address this clinical problem, researchers need to identify new chemotherapeutic drugs that can be derived from readily available sources and have fewer and less severe side effects. At present, natural anticancer products have attracted increasing attention due to their few side effects and wide availability. Some studies have confirmed that polyphenols in green tea leaves can prevent and mitigate cancer progression [5–7]. Catechins are the primary polyphenols in green tea and have various biological activities. Epigallocatechin-3-gallate (EGCG) comprises approximately 50%–80% of catechins in green tea and has the strongest biological activity among these components [8].

EGCG is a flavanol, and its molecular structure indicates that it is strongly hydrophilic and has antioxidant and cell membrane penetration properties [9,10]. Numerous studies have shown that EGCG exerts powerful antitumour effects through its antioxidant activities to inhibit tumor growth and tumor angiogenesis and promote tumor cell apoptosis, and these effects have been reported in malignant tumors of various organs, including the liver, lung and prostate [11–13]. Furthermore, many studies have demonstrated that EGCG can prevent tumor growth and inhibit angiogenesis and tumor metastasis by regulating various signaling pathways, including the p38/MAPK [14], PI3K/Akt [15], and Wnt/β-catenin [16] pathways, which are closely related to cell proliferation, apoptosis, and migration in tumors. However, the effect of EGCG on OS cells and the definite mechanisms involved in this effect have not been fully elucidated.

However, the effect of EGCG on OS cells and the definite mechanism involved still need to be further explored. We envisage that EGCG also has anti-tumor properties on OS, the specific mechanism of its anti-tumor effect was further clarified by conducting cellular and animal experiments in this research, in order to find a new natural anti-osteosarcoma drug. First, based on MTT assays and crystal violet staining of the 143B, MG63, and SaOS2 human OS cell lines, we found that the proliferation of OS cells was substantially inhibited by EGCG. Among these cell lines, 143B showed the most stable and sensitive response to EGCG and were thus selected for the next experiments. We found that EGCG strongly suppressed 143B cell invasion and migration and significantly promoted apoptosis. Furthermore, EGCG affected the expression of proliferation-, apoptosis- and migration-related proteins. In our in vivo experiments, the growth of OS was significantly inhibited by EGCG in a dose-dependent manner, and this treatment significantly inhibited lung metastasis. In addition, we found that the effect of EGCG was closely related to the Wnt/β-catenin pathway. The overexpression of knockdown of β-catenin strengthened or weakened the antitumour effect of EGCG, respectively. Therefore, our study indicated that the antitumour effect of EGCG is closely related to the Wnt/β-catenin signaling pathway, and this molecule is thus a new research hotspot and a promising antitumour drug for the treatment of OS.

## Materials and methods

### Cell culture and drug preparation

Three types of human OS cell lines, MG63, 143B and SaoS2 (America Type Culture Collection, USA), were examined by STR analysis. All experimental cells were cultured in complete Dulbecco’s modified Eagle’s medium (DMEM, Grand Island, NY, USA) containing 1% penicillin, 1% streptomycin and 10% fetal bovine serum (FBS, Grand Island, NY, USA) at 37°C in a humidified incubator with 5% CO_2_. EGCG (purity>98%, Chengdu Herbpurify Co., Ltd., Chengdu, China) was dissolved with DMSO (Dimethyl sulfoxide) and uniformly mixed to generate a 100 mg/ml stock solution. After filter sterilization (0.22 µm), the filtered stock solution was aliquoted, stored at −20°C and diluted with DMEM to a specific treatment concentration before each use.

### Crystal violet viability assay

Crystal violet staining was performed as described previously [17]. In brief, when the confluence of OS cells in 24-well plates reached approximately 30%, the incubation was continued for 24, 48, and 72 hours after treatment with EGCG at different concentrations. The cells were then washed with phosphate-buffered saline (PBS), fixed with 4% paraformaldehyde at 37°C for 10 minutes and again washed with PBS. The cells were subsequently stained with Giemsa dye for 10 minutes, repeatedly soaked in water until the dye solution was rinsed out, dried in an oven with a constant temperature of 37°C and photographed. For quantitative determination, the cells were then lysed with 10% acetic acid solution (1 ml per well) at room temperature with continuous shaking for 20 minutes. A 500-μl aliquot of the cells was diluted with 2 ml of double-distilled H_2_O to measure the absorbance at 570 to 590 nm.

### Hoechst staining test

When the OS cells in the 24-well plates reached 60%–70% confluence, the cells were treated with EGCG. After treatment with EGCG for 24 hours, the cells were gently washed three times with PBS and then fixed with 4% paraformaldehyde for 10 minutes at 37°C. After the paraformaldehyde was discarded, the cells were gently washed three times with PBS and then stained with Hoechst 33,258 dye for 5 minutes in the dark [18]. The nuclear morphology was analyzed by fluorescence microscopy (Eclipse Ti, Nikon, Japan).

### MTT assay

The cell viability was analyzed by MTT [3-(4,5-dimethylthiazol-2-yl)-2,5-diphenyl-tetrazolium bromide] colorimetric assays. A total of 3000 cells per well were seeded in a 96-well plate and treated with various concentrations of EGCG or DMSO for 0, 24, 48, and 72 hours. Subsequently, 20 µl of MTT reagent (5 mg/ml; Sigma–Aldrich, St. Louis, MO, USA) was added to each well, and the plate was incubated for 4 hours in an incubator at 37°C. After the cell culture medium was discarded, 150 µl of DMSO was added to each well, and the plate was incubated for 15 minutes. A microplate reader was then used to detect the absorbance value of the culture medium at 492 nm. The OD values at different time points were converted using the blank group value as the 100% reference point, and the converted results were used for statistical analysis of cell proliferative activity [18].

### Flow cytometry

When the confluence of 143B cells in 6-well plates reached approximately 60%, the cells were treated with different concentrations of EGCG. After treatment with EGCG for 24 hours, the cells were digested and collected. The collected cells were thoroughly washed with cold PBS, centrifuged, fixed with 70% alcohol, and stored overnight in a refrigerator. 143B cells in the logarithmic phase of growth were seeded in 6-well plates. After treatment with EGCG for 24 hours, the cells were digested, washed with PBS and fixed with 70% ethanol overnight at 4°C. The processed cells were then reacted with PI/Triton X-100 for 15 minutes. The cell cycle and cell apoptotic distribution were measured with a CytoFLEX flow cytometer (Beckman Coulter, Fullerton, CA, USA) as described previously [19].

### Cell apoptosis assay

First, 143B cells were inoculated in 6-well plates, and after the cell confluence reached 60%, the cells were treated with EGCG for 24 hours. The cells were collected at the designated time, washed repeatedly with PBS, and then resuspended in 500 µl of PBS [18]. The distribution of cells in early and late apoptosis was detected by flow cytometry using an Annexin V-FITC/PI double labeling staining kit (Beyotime Biotechnology, Shanghai, China).

### Transwell migration assay

The upper Transwell chamber (Corning Corporation, USA) was seeded with approximately 20,000 cells per well and 200 µl of culture medium with 10% FBS, and 600 µl of culture medium (10% FBS) containing different concentrations of EGCG was added to the lower Transwell chamber. After culture for 24 hours, the cells that migrated to the lower chamber were fixed with 4% paraformaldehyde and then stained with 0.1% crystal violet [18].

### Matrigel invasion assays

Matrigel (Millipore, MA, USA) was diluted from 5 mg/ml to 1 mg/ml with serum-free cold cell culture medium (DMEM), and 100 µl was added to the upper chamber of the Transwell. The Transwells were placed in an oven at a constant temperature of 37°C for approximately 2 hours until gels formed. The next experimental steps were the same as those in the Transwell migration assay [18].

### Colony forming assay

Approximately 1,000 143B cells were inoculated in a 6-well plate and cultured with 1% FBS medium until the cells adhered to the wall, and EGCG at different concentrations was then added. After 7 days, the cells were stained with 0.1% crystal violet staining solution for 10 minutes [18]. The number of colonies was determined using ImageJ software (≥50 cells/colonies was defined as a colony).

### Wound-healing assay

Cells in 6-well plates were treated with EGCG after they reached 90% confluence. The cells were then wounded with 10-µl pipette tips and cultured in serum-free medium. Photos of the wound area were taken 0, 12, and 24 hours after wounding. The wound-healing rate was calculated as follows: (0-hour width – 12-hour or 24-hour width)/0-hour width × 100% [18].

### Transfection and luciferase reporter assay

The protocol for the firefly luciferase reporter assay was described previously [18]. Briefly, 3.0 g of TCF/LEF luciferase reporter was added to culture flasks, and transfection was performed with Lipofectamine^TM^ 2000 (Invitrogen, Gaithersburg, MD, USA). Twenty-four hours after transfection, the treated cells were replanted in 24-well plates, and EGCG or DMSO was then added to the cells in each group. After 24 hours, the upper medium was collected for fluorescence activity detection using a Gaussia Luciferase Assay kit (New England Biolabs).

### Western blotting

First, the cells were washed with cold PBS and lysed using RIPA lysis buffer (Beyotime, Haimen, China) containing protease and phosphatase inhibitors. The protein concentrations were measured using a bicinchoninic acid (BCA) protein assay kit (Beyotime, Haimen, China). Equal amounts of protein from different groups were separated by sodium dodecyl sulfate–polyacrylamide gel electrophoresis (SDS–PAGE). For immunoblot analysis, proteins were transferred to polyvinylidene difluoride (PVDF) membranes and blocked with Tris-buffered saline/Tween 20 (TBST) with 5% bovine serum albumin (BSA; Solarbio, Beijing, China) at 37°C for 2 hours. The PVDF membranes were then incubated with β-actin antibody (Abcam, MA, USA) or other primary antibodies (Cell Signaling Technology, Danvers, MA, USA) at 4°C overnight, shaken, washed three times with TBST and incubated with secondary antibodies (1:5000; ZhongShan Golden Bridge, Beijing, China) for 1 hour at 37°C. Western blot development was performed using BeyoECL Plus (Beyotime, Haimen, China), as described previously [19]. The ChemiDoc MP Imaging System was purchased from Bio–Rad (Bio–Rad, California, USA).

### Construction of an animal model of ectopic OS

The proximal femur periosteum of 4- to 6-week-old female nude mice was injected with 50 µl (2 × 10^7^ cells/ml) of 143B cells. The mice in the experimental groups and the control group were given EGCG (10, 20 or 40 mg/kg) and the control treatment (sodium carboxymethyl cellulose, CMC-Na), respectively, once every 2 days. The size of the tumor tissue was recorded every 2 days starting from the second week after tumor cell injection. Twenty-one days after tumor cell injection, the mice were sacrificed by cervical dislocation, and death was determined based on breathing and heartbeat. The tumor size was calculated as follows: 0.5 × L× W^2^ (L represents the tumor length, and W represents the tumor width; the tumor tissue size did not exceed 2 cm). Tumor tissue, lung tissue and bone specimens were recovered for histological examination [18]. All animal experiments were approved by the Institutional Animal Care and Use Committee (IACUC) of Chongqing Medical University the First Affiliated Hospital of Chongqing Medical University.

### TUNEL assay

Terminal deoxynucleotidyl transferase dUTP nick-end labeling (TUNEL) assay was performed using Colorimetric TUNEL Apoptosis Assay Kit (Beyotime, Haimen, China) according to manufacturer’s protocol. Briefly, after deparaffinization, gradient hydration, and PBS washing, the tumor sections were treated with proteinase K (without DNase) to permeabilize the cells, washed again with PBS, and fixed with paraformaldehyde. Then, tumor sections were incubated in 3% H2O2 and then in the TUNEL reaction mixture. The sections were rinsed and visualized using diaminobenzidine. Hematoxylin was used for counterstaining. The cell apoptosis rate was calculated as the percent of TUNEL positive cells relative to the total cells [20].

### Immunohistochemistry (IHC)

The recovered tumor and lung tissues were first immersed and fixed in paraformaldehyde and then embedded in paraffin. The retrieved bone tissues were completely decalcified with ethylenediaminetetraacetic acid (EDTA) decalcification agent (Beijing Solarbio Science and Technology Co., Ltd., Beijing, China) for 2 weeks, and the specimens were then embedded in paraffin and sectioned into serial sections with a thickness of 4 mm. For histological analysis, the tissue sections were deparaffinized and stained with hematoxylin and eosin (H&E). The tumor tissue sections were blocked and immunostained with antibodies targeting Bcl-2 (1:100), vimentin (1:50), PCNA (1:100) and β-catenin (1:100). The primary antibodies, including anti-Bcl-2, vimentin and PCNA antibodies, were obtained from Cell Signaling Technology (CST; Danvers, MA, USA). An inverted microscope was used to capture randomly selected images, and analysis of the IHC images was performed with Image-Pro Plus 6.0 software [18].

### Statistical analysis

The experimental data were analyzed using SPSS 18.0 statistical software (SPSS, Inc., Chicago, IL, USA). The statistical data are presented as the means ± SDs or means ± SEMs (n = 3, each group). All experiments were effectively repeated. Comparisons between groups were performed using one-way ANOVA followed by Tukey’s post-hoc test. A significant difference was defined by *p* < 0.05.

## Results

In this study, in order to explore the effect and specific mechanism of EGCG on OS. We found that EGCG can not only inhibit the proliferation, migration and invasion of OS cells, but also promote their apoptosis. Moreover, further experimental results confirmed that the anti-osteosarcoma growth effect of EGCG is achieved by regulating the Wnt signaling pathway. In addition, the nude mouse ectopic OS model further confirmed that EGCG can inhibit the growth of OS, protect the orthotopic bone from tumor destruction, and inhibit the lung metastasis of OS.

## EGCG inhibits OS cell proliferation

**Figure 1.**
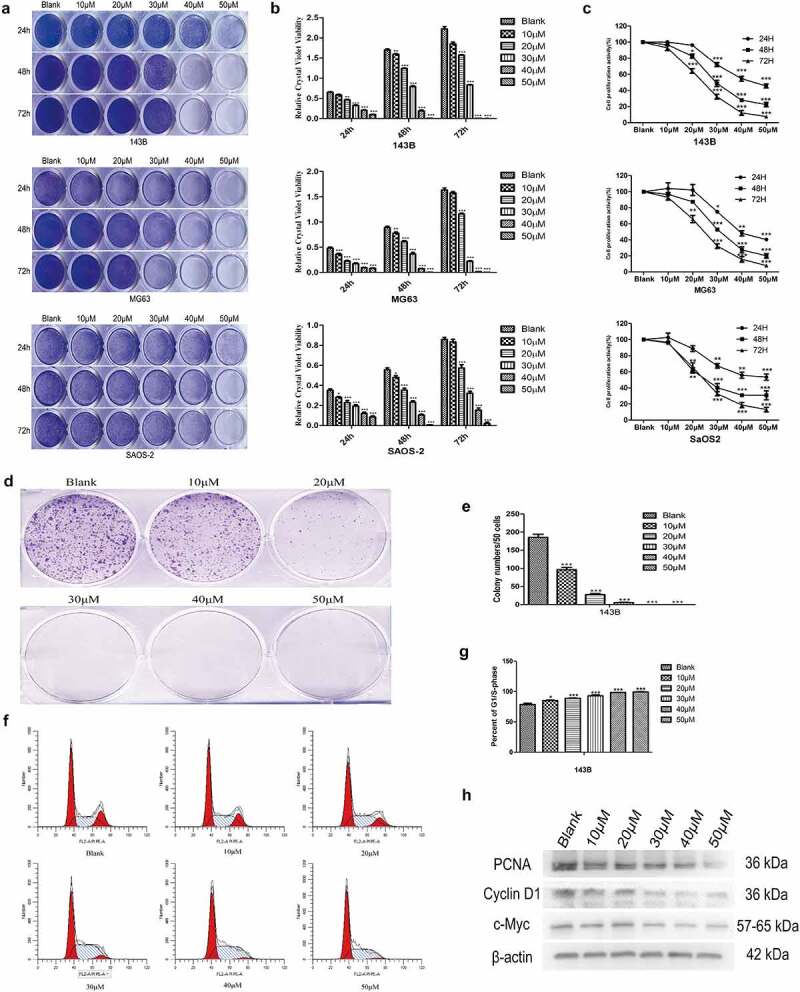
EGCG inhibits OS cell proliferation. The crystal violet staining (a, b), MTT assay (c) and colony formation assay (d, e) results revealed the influence of EGCG on human OS cell proliferation. (f, g) The effect of EGCG on the OS cell cycle distribution was detected by flow cytometry after treatment. (h) After treatment with EGCG for 3 days, the expression of proliferation-related proteins was detected by Western blotting. *p < 0.05, ** *p* < 0.01, and *** *p* < 0.001 vs. the blank group.

As described previously, some scholars have shown that EGCG can affect the growth of various tumors. MTT assays and crystal violet staining revealed that the proliferation of 143B, MG63 and SaOS2 OS cells was suppressed to a greater degree in the EGCG-treated groups than in the control group (Figure 1a–c). The results from the MTT and crystal violet experiments revealed that 143B cells showed more sensitive and stable results than MG63 and SaOS2 cells; thus, 143B cells were used in all subsequent experiments. Colony formation experiments indicated that the cells treated with EGCG, particularly those treated with the high dose of EGCG, formed substantially fewer and smaller colonies than the control cells (Figure 1d,e). Moreover, the flow cytometric results revealed that after EGCG treatment, most of the 143B cells were arrested at the G1/S transition (Figure 1f,g). The treatment of OS cells with EGCG also notably inhibited the expression of proliferation-related proteins, including cyclin D1, PCNA and c-Myc, compared with the levels found in the control group (Figure 1h). These results show that the proliferation of OS cells is substantially inhibited by EGCG and that this inhibitory effect may be dependent on both time and concentration.

### EGCG promotes OS cell apoptosis

**Figure 2.**
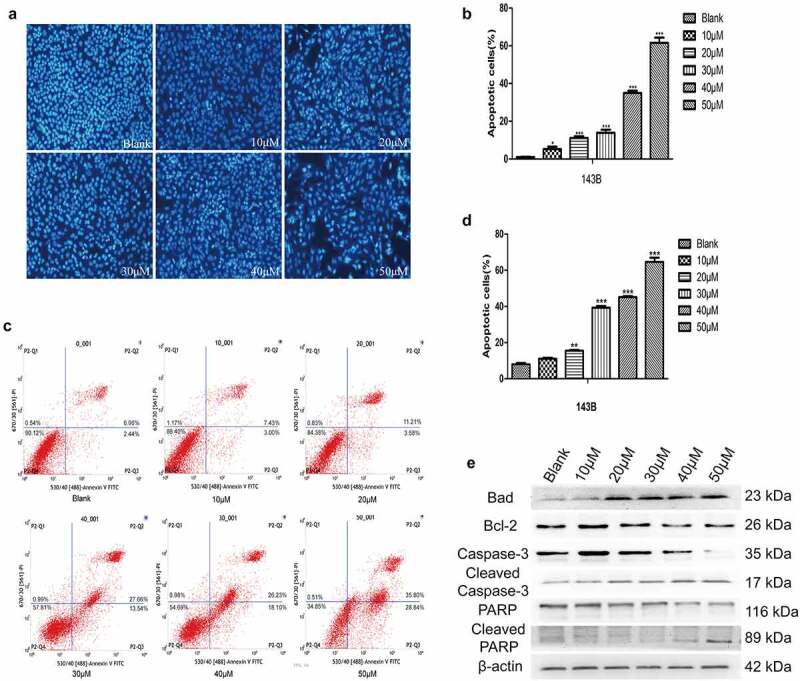
EGCG promotes OS cell apoptosis. (a, b) The apoptosis of 143B cells was detected by Hoechst staining. Magnification, ×100. (c, d) The apoptosis of 143B cells was detected by flow cytometry. (e) After treatment with EGCG for 3 days, the expression of apoptosis-related proteins was detected by Western blotting. *p < 0.05, ** *p* < 0.01, and *** *p* < 0.001 vs. the blank group.

In addition to inhibiting the proliferation of tumor cells, antitumour drugs should also promote apoptosis. The Hoechst staining and flow cytometric results showed that the apoptosis of 143B cells was significantly induced to a greater degree in the experimental groups treated with different concentrations of EGCG than in the control group, and the difference between the groups was significant (Figure 2a–d). In addition, the expression of cleaved caspase-3, Bad and cleaved PARP, which are apoptosis-related proteins, was significantly increased after treatment with EGCG, whereas the expression of Bcl-2, which is a negative regulator of apoptosis, was strongly inhibited in the EGCG-treated groups (Figure 2e). The above results indicate that the ability of EGCG to promote apoptosis of 143B cells gradually increases with increases in the treatment concentration, and the optimal concentration for inducing apoptosis was found to be approximately 30–40 µM.

### EGCG suppresses the invasion and migration of OS cells

**Figure 3.**
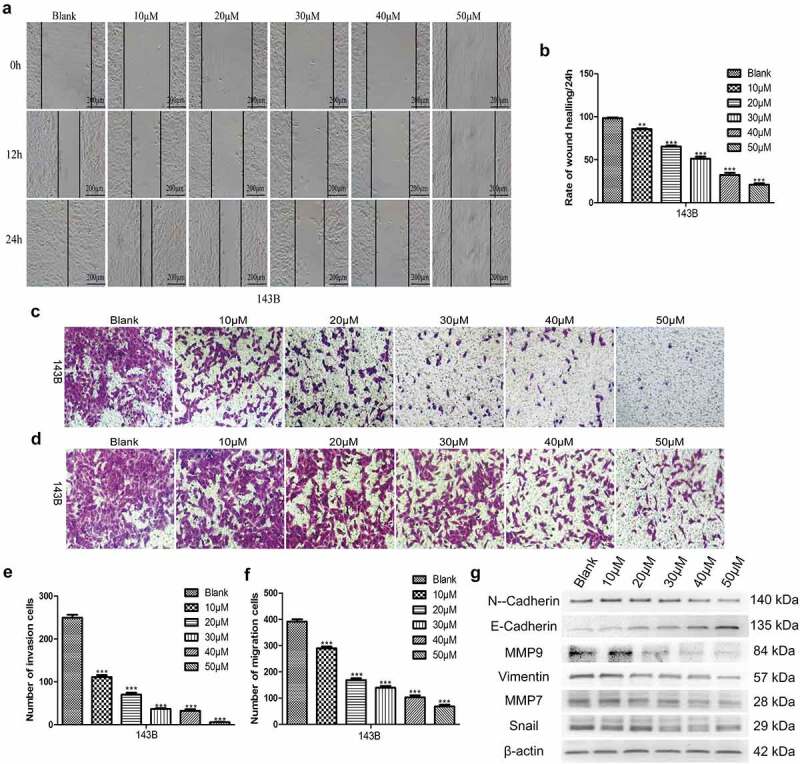
EGCG suppresses the metastatic ability of 143B cells. (a, b) The migratory capability of cells after treatment with EGCG for 0, 24 and 72 hours was detected by wound-healing tests. Magnification, ×100. The invasion (c, e) and migration (d, f) of 143B cells were detected by Transwell assays. Magnification, ×100. (g) After treatment with EGCG for 3 days, the expression of migration- and invasion-related proteins in 143B cells was detected by Western blotting. *p < 0.05, ** *p* < 0.01, and *** *p* < 0.001 vs. the blank group.

Preventing malignant tumors from invading surrounding tissues and metastasizing to distant organs is a highly desirable characteristic of antitumour drugs. Therefore, we assessed the invasion and migration of 143B cells after treatment with EGCG. The wound-healing and Transwell migration assay results showed that EGCG strongly inhibited the migratory ability of 143B cells, and the Matrigel invasion assays also indicated that the invasive capacity was strongly suppressed after treatment with EGCG (Figure 3a–f). We then evaluated the expression of migration- and invasion-associated proteins and found that the expression of these proteins was strongly suppressed after treatment with EGCG, which is consistent with the above-described experimental results (Figure 3g). These findings indicate that EGCG effectively suppresses the invasion and migration of OS cells in vitro and shows potential for antitumour treatment.

### EGCG inhibits OS growth by regulating the Wnt/β-catenin pathway

**Figure 4.**
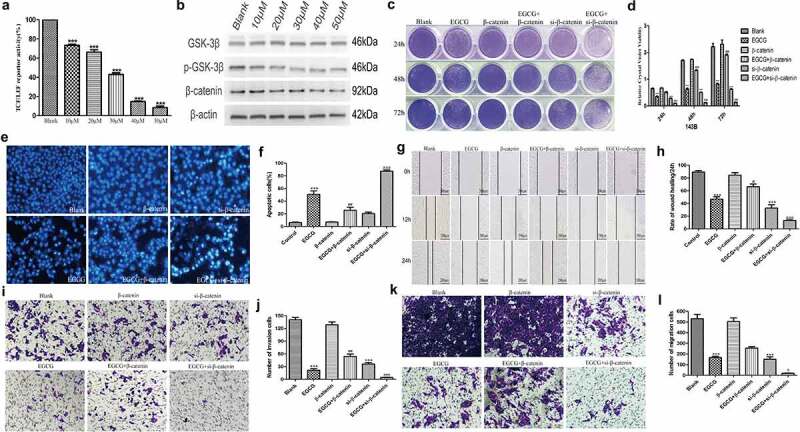
EGCG regulates the Wnt/β-catenin pathway to exert an anti-OS effect. (a) After treatment with EGCG for 24 hours, the activity of the TCF/LEF-Luc reporter was analyzed. (b) The expression of β-catenin after treatment with EGCG for 3 days was detected by Western blotting. (c, d) Regulation of Wnt/β-catenin pathway activity affected the inhibitory effect of EGCG on tumor growth, as shown by crystal violet staining. (e, f) Regulation of Wnt/β-catenin pathway activity influenced the effect of EGCG on tumor apoptosis, as shown by Hoechst staining. (g–l) Regulation of Wnt/β-catenin pathway activity influenced the effect of EGCG on tumor invasion and migration, as demonstrated by wound-healing tests and Transwell assays, respectively. *p < 0.05 vs. the blank group, ^#^*p* < 0.05 vs. the EGCG+β-catenin group, ^a^*p* < 0.05 vs. the EGCG+si-β-catenin group.

In this study, the specific mechanism through which EGCG exerts its anti-OS effects was further explored. First, 143B cells were transfected with multiple fluorescent reporter plasmids and treated with different concentrations of EGCG, and the results showed that the activity of the TCF/LEF-Luc reporter was significantly inhibited (Figure 4a). Western blotting results showed that treatment with EGCG notably suppressed the expression of β-catenin, which is the key protein of the Wnt signaling pathway, in OS cells (Figure 4b). To further demonstrate the importance of the Wnt/β-catenin pathway in the antitumour effect of EGCG on OS cells, we regulated β-catenin expression by knocking it down with siRNA or overexpressing an adenoviral plasmid containing the β-catenin coding sequence and then observed the effects of these changes on the antitumour activity of EGCG. Crystal violet staining of the resulting cells showed that β-catenin overexpression significantly inhibited the antitumour effect of EGCG and that β-catenin knockdown strengthened the antitumour effect of EGCG (Figure 4c,d). The results of Hoechst staining indicated that the ability of EGCG to promote OS cell apoptosis was inhibited in β-catenin-overexpressing cells and that interfering with the expression of β-catenin strengthened the ability of EGCG to promote OS apoptosis (Figure 4e,f). Subsequently, Transwell migration, Matrigel invasion and wound-healing assays confirmed that EGCG treatment promoted tumor cell migration and invasion in β-catenin-overexpressing OS cells, but the downregulation of β-catenin expression significantly inhibited the ability of tumor cells to metastasize (Figure 4g–l). The above-described results strongly indicate that the growth of OS cells is substantially inhibited by EGCG through regulation of the Wnt/β-catenin signaling pathway.

### EGCG inhibits the growth of OS in vivo

**Figure 5.**
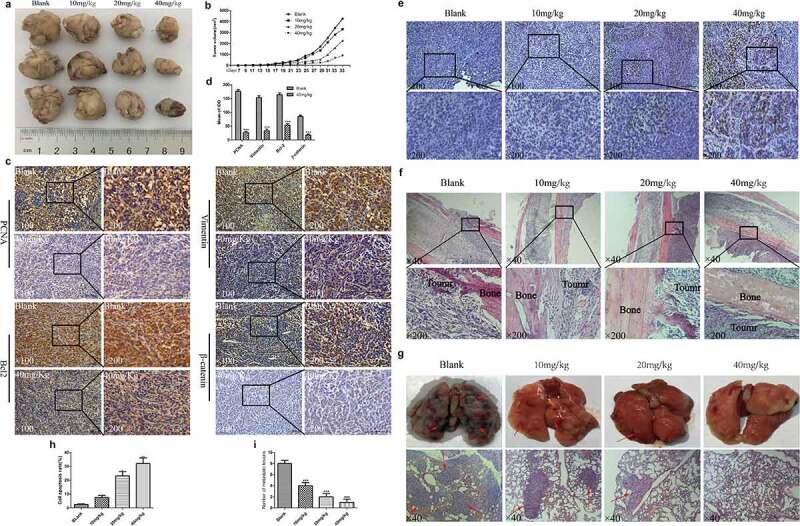
EGCG inhibits OS growth in vivo. (a, b) Macroscopic view of recovered tumor tissues and graph showing tumor growth. (c, d) The expression of PCNA, vimentin, Bcl-2 and β-catenin in tumor tissues was detected by IHC. Magnification, ×100 and ×200. (e, h) Detection of apoptosis in tumor tissue by Tunel assay. Magnification, ×100 and ×200. (f) H&E staining of bone tissue at the tumor cell injection site. Magnification, ×40 and ×200. (g, i) Macroscopic view of recovered lung tissue and H&E staining of tissue sections. The red arrow points to the tumor metastasis. Magnification, ×40. *p < 0.05, ** *p* < 0.01, and *** *p* < 0.001 vs. the blank group.

The antitumour effect of EGCG was further verified through animal experiments. A tumor model with 143B cells in nude mice was established. Our results showed that the recovered tumor tissues were clearly smaller in the different EGCG treatment groups than in the blank group, and this inhibition was concentration dependent; this finding is similar to the results from the in vitro experiments (Figure 5a,b). Further immunohistochemical results indicated that the expression levels of PCNA, the antiapoptotic regulator Bcl2, the migration-related protein vimentin, and the Wnt pathway protein β-catenin were significantly reduced in tumor tissues (Figure 5c,d). Moreover, the Tunel experiment further confirmed that EGCG can significantly promote the apoptosis of osteosarcoma cells, and this effect is obviously dose-dependent (Figure 5e,h). After recovering the specimen and H&E staining of the area of the femur close to the tumor xenograft, we found that the control and low-dose treatment groups showed obvious local invasion and damage to the bone tissue, whereas the high-dose treatment group showed no invasion or damage to the bone tissue (Figure 5f). Based on the appearance and H&E staining of the recovered lung tissue, we found different degrees of metastasis in the control and low-dose treatment groups; specifically, the lung tissue was notably destroyed in the absence of EGCG treatment (Figure 5g,i). In contrast, the lung tissue from the high-dose treatment group was essentially normal (Figure 5g,i). These results indicate that EGCG can suppress OS growth in vivo while mitigating damage to bone tissue and metastasis to the lungs.

## Discussion

The existing treatment options for OS have stagnated with regard to improving the long-term prognosis of patients, and most of the existing radiotherapy and chemotherapy regimens often result in serious adverse reactions. Therefore, the identification of a safe and effective anti-OS drug (preferably derived from natural products) that can be combined with surgical treatment is urgently needed. Many previous studies have reported that components in green tea can prevent cardiovascular disease, diabetes, cancer and other diseases [21]. Among the various catechins in green tea, EGCG exhibits the highest content and activity. Many studies have also confirmed that EGCG has anti-inflammatory, antioxidant, antiangiogenic, antiproliferative, proapoptotic, and antimetastatic activities and can alleviate drug resistance [21–23]. However, few studies have investigated the effects of EGCG on OS, and the specific mechanism involved is unclear. Therefore, we performed cell and animal experiments to determine how EGCG affects OS cell growth and clarified the specific antitumour mechanism of EGCG by overexpressing or downregulating the expression of β-catenin. Some scholars have interfered with the expression of TGF-β1 to confirm that EGCG inhibits the epithelial transformation and invasion of thyroid cancer cells by regulating the TGF-β1-Smad4 pathway. Compared with the above studies, the cell lines used in our experiment are more relatively complete, and clarified the specific antitumour mechanism of EGCG through over-expressing or down-regulating the expression of β-catenin and animal experiments, and these methods make the findings more convincing.

In previous studies, the different concentrations of EGCG used for in vivo experiments were in the range of 10 to 100 mg/kg, and the doses used for in vitro experiments were in the range of 0 to 100 µM [21]. Our crystal violet staining and MTT assay results showed that EGCG at low concentrations could notably decrease the cell viability of three OS cell lines, and the half-maximal inhibitory concentration (IC_50_) of EGCG was approximately 30 µM. Therefore, we chose 10 µM, 20 µM, 30 µM, 40 µM and 50 µM EGCG as the experimental treatment concentrations for our subsequent experiments. Unlimited proliferation is one of the main characteristics of malignant tumor cells. Our flow cytometric results from this study confirmed that most of the cells were blocked at the G1/S transition after treatment with EGCG, and the expression of proliferation-related proteins was obviously reduced. These findings are similar to previous research results [21,24]. The experimental results at this stage show that EGCG can significantly suppress the proliferation of tumor cells and that the possible mechanism involved is related to the influence of EGCG on the mitochondrial membrane potential [25].

Antitumour drugs should ideally counteract the abnormal apoptosis, invasion and migration of malignant tumor cells [26], and these conditions are often related to abnormal protein expression, increased oxidative stress or abnormal activity of signaling pathways, such as the MAPK/Erk [27], NF-κB [28] and PI3K/Akt [15] pathways. Our results showed that the metastatic ability of OS cells was effectively inhibited by EGCG, which also promoted apoptosis. Furthermore, the expression of proteins associated with tumor cell metastasis, including MMP7, MMP9, N-cadherin, vimentin and Snail, was significantly reduced, and the corresponding expression of proteins related to tumor cell apoptosis, such as caspase-3, PPAR and GSK-3β, was strongly increased. However, Bcl-2 is a regulatory factor that inhibits apoptosis, and its expression was clearly inhibited in the EGCG-treated groups. Previous studies have demonstrated that the highly active Wnt/β-catenin pathway (particularly the abnormal expression and localization of β-catenin) is an indispensable precursor for the occurrence and development of tumors and is related to various diseases [29]. In our research, we found that EGCG could significantly affect the activity of the Wnt/β-catenin pathway based on alterations in the fluorescence of 143B cells transduced with a fluorescent reporter plasmid after treatment with EGCG. Through both cell and animal experiments, we then found that β-catenin was significantly suppressed at the protein expression level and that this inhibition was positively correlated with the concentration of EGCG. In addition, by overexpressing or knocking down β-catenin, we found that EGCG exerts anti-OS effects by regulating the Wnt/β-catenin signaling pathway.

Moreover, the influence of EGCG on OS in situ and the prevention of pulmonary metastasis were further investigated through animal experiments. During the feeding period, the tumor growth rate after treatment with EGCG, particularly the high dose, was clearly slower than that found in the control group. A macroscopic analysis of the recovered tumors revealed no notable difference in tumor size between the blank group and the low-dose treatment groups, which may be related to the low bioavailability of EGCG in vivo [30]. To further evaluate the apoptosis of cells in tumor tissues, we found by the TUNEL experiment that only a few cells appeared apoptosis in the tumor tissues of the control group and the low-dose EGCG treatment group. With the increase of EGCG dose, the number of apoptosis increased significantly, which was consistent with the results of cell experiments and immunohistochemistry. Further histological results suggested that the synthesis of PCNA and β-catenin was clearly affected by EGCG in tumor tissues, and both of these proteins are closely linked to the ability of tumor cells to proliferate indefinitely [31], which may explain the inhibition of tumor growth observed in the EGCG-treated groups. These results show that EGCG can significantly inhibit the growth of osteosarcoma cells and promote their apoptosis, and this anti-tumor effect has obvious effects in both in vitro and in vivo experiments.

Local invasion and early lung metastasis are two main factors that are closely related to the unsatisfactory prognosis of OS [32]. In this study, the general assessment of the recovered lung tissue showed that the metastasis of OS cells into the lung tissue of the blank group was severe, whereas the lung tissues of the EGCG-treated groups were well protected and showed no obvious tumor metastasis or destruction; notably, the lung tissue destruction in the high-dose treatment group was less severe than that in the other groups. Furthermore, the results of the H&E staining of lung tissues from each group was consistent with these findings. We also analyzed the invasion of OS cells into local bone tissues and adjacent joints via H&E staining, and the results for the control group showed that OS cells significantly invaded and destroyed the local bone tissue, some tumor cells spread into the medullary cavity, and adjacent joints were damaged to varying degrees. However, in the EGCG-treated groups, the local bone tissue and adjacent joints were protected to a certain extent, and this protective effect was positively correlated with the concentration of EGCG.

## Conclusion

We confirmed that EGCG can effectively inhibit the tumor characteristics of OS cells, including proliferation, migration, and apoptosis, while protecting local bone tissue from destruction and preventing lung metastasis of tumor cells, and this effect may occur by regulating the activity of the Wnt/β-catenin pathway, as demonstrated by cell and animal experiments. These outcomes indicate that EGCG exerts a significant effect on the treatment of OS and can be used as a potential OS treatment or chemotherapeutic drug. However, our data do not account for the heterogeneity of OS tumors, and future research is needed to address the low bioavailability of EGCG and elucidate the synergistic effect(s) of EGCG and other antitumour drugs.

## Data Availability

The experimental data can be obtained within the scope of reasonable requirements from the corresponding author.
